# Fertility Alteration Characteristics and Cytological Mechanisms of Pollen Abortion in Thermo-Photo-Sensitive Genic Male Sterile Wheat K64S

**DOI:** 10.3390/plants15121774

**Published:** 2026-06-09

**Authors:** Hongsheng Li, Xiong Tang, Zhonghui Yang, Jian Yin, Shaoxiang Li, Kun Liu, Mingliang Ding, Yao Tang, Munjun Yang, Xiue Wang

**Affiliations:** 1 Food Crops Research Institute, Yunnan Academy of Agricultural Sciences, Kunming 650205, China; lhs@yaas.org.cn (H.L.); txynnkylzs@163.com (X.T.); yzh@yaas.org.cn (Z.Y.); yinjian199605@163.com (J.Y.); lsx@yaas.org.cn (S.L.); lk@yaas.org.cn (K.L.); dml@yaas.org.cn (M.D.); km2026tang@163.com (Y.T.); 2 State Key Laboratory of Crop Genetics and Germplasm Enhancement, College of Agriculture, Cytogenetics Institute, Nanjing Agricultural University, Nanjing 210095, China

**Keywords:** hybrid wheat, thermo-photo-sensitive genic male sterile line, fertility alteration, pollen abortion, tapetal degeneration

## Abstract

This study identified the fertility alteration characteristics and cytological mechanisms of the thermo-photo-sensitive genic male sterile (TPSGMS) wheat line K64S. The fertility-sensitive stage of K64S extends from pollen mother cell formation to the tetrad development stage, with critical fertility alteration thresholds of 14–14.5 °C for temperature and 9–9.5 h for daylength. Under low-temperature and short-day conditions, K64S exhibits complete male sterility, whereas it returns to fertility under high-temperature and long-day conditions. Cytological analysis shows that K64S undergoes normal meiosis and successfully forms normal uninucleate microspores. 4′,6-diamidino-2-phenylindole (DAPI) staining revealed the uninucleate microspores failed to form binucleate microspores, with abortion occurring during the late uninucleate stage. Transmission electron microscopy indicates the pollen abortion in sterile K64S arises primarily from premature tapetal degeneration (a form of programmed cell death, PCD), initiated at the pollen mother cell stage, which disrupts nutrient supply and leads to abnormal nuclear division during subsequent microspore development. These findings provide insights into the cytological mechanism of pollen abortion in TPSGMS wheat and may guide hybrid wheat breeding and application.

## 1. Introduction

With the global population projected to reach 9.7 billion by 2050, a 2.4% annual increase in wheat production is essential. However, current yield growth rates in major wheat-producing regions have slowed to 1% (FAO, 2023), underscoring the urgent need for effective interventions. Hybrid wheat breeding offers opportunities to enhance yield potential, especially in marginal environments [1,2,3,4]. Hybrid wheat yields can exceed those of the best inbred commercial cultivars by 10 to 20%, particularly in saline and rainfed fields [5,6,7,8,9]. Furthermore, hybrid cultivars outperform inbred lines in drought tolerance, nutrient use efficiency, and root system development [10], while demonstrating better adaptation to both biotic and abiotic stresses [6].

In autogamous crops such as wheat, the induction of male sterility to prevent self-pollination is a crucial strategy for hybrid seed production. Several two-line systems for inducing male sterility have been developed, including genic male sterility (GMS) [11,12,13,14,15,16,17], environment-sensitive genic male sterility (thermo-photo/photo-thermo/thermo-sensitive male sterility and cytoplasmic male sterility) [18,19,20,21,22], as well as chemical hybridization agents (CHAs) [23,24]. Additionally, three-line systems based on cytoplasmic male sterility (CMS) have been effectively utilized in hybrid wheat breeding programs [25,26,27]. Worldwide, numerous hybrid wheat cultivars have been launched, and 33 cultivars developed from two-line system of photo-thermo- or thermo-photo-sensitive male sterility have been released in China [28]. However, the large-scale application of hybrid wheat remains limited, constituting merely 1% of the global wheat production area. The primary obstacle is the lack of an economically viable hybridization system that can produce hybrid seeds at low cost in commercial applications [4,5,12].

The three-line system requires a two-phase seed production process: (1) sterile line multiplication by cross-pollination between the sterile line and its maintainer and (2) hybrid seed production by cross-pollination between the sterile line and its restorer. Moreover, the limited availability of restorer germplasm significantly constrains hybrid yield potential and increases production costs, impeding large-scale application [5]. While CHAs offer greater flexibility in parental combinations and simplify the hybrid seed production system, however, the shortage of stable and environmentally friendly CHAs, together with the vulnerability of male sterility effects to weather conditions, varietal differences, and application timing of CHAs, often leads to poor seed quality and even complete production failures [29,30]. In contrast, the environment-sensitive two-line system, which includes thermo-photo-sensitive genic male sterile (TPSGMS) and photo-thermo-sensitive genic male sterile systems, has emerged as the predominant methodology in Chinese hybrid wheat breeding. This approach benefits from extensive restorer germplasm resources and streamlined seed production processes [4,8,9,18,28]. Investigations have addressed outcrossing characteristics of TPSGMS lines, enhanced outcrossing ability of restorers (e.g., anther exsertion), and optimized hybrid seed production methods, culminating in hybrid seed yields of up to 4500 kg/ha in preliminary commercial application [31,32,33,34,35,36,37]. However, the fertility of TPSGMS lines is not only controlled by unknown nuclear recessive genes but also strictly regulated by temperature and photoperiod, with sterility under low-temperature/short-day conditions and fertility under high-temperature/long-day conditions [18]. Precise characterization of fertility alteration parameters, including critical temperature/photoperiod thresholds, sensitive developmental stages, and sterility mechanisms, is therefore essential for TPSGMS line propagation and hybrid seed production, as well as for investigation of molecular basis next step.

Previous studies have shown that TPSGMS lines exhibit distinct fertility alteration patterns. C49S, the first reported TPSGMS line in China, shows maximal fertility sensitivity during pollen mother cell meiosis and the mid-microspore stage, with complete sterility thresholds of 8.5 °C (mean minimum temperature) during meiosis and 13.5 °C (mean temperature) and 10.5 °C (mean minimum temperature) during the mid-microspore stage; nearly normal fertility is observed at thresholds of 11.5 °C, 15.0 °C, and 12.5 °C, respectively [38]. However, several C49S-derived lines, including K78S, MTS-1, and C412S, exhibit substantially different fertility alteration parameters [39,40,41]. These findings indicate substantial variation even among lines sharing common ancestry, a phenomenon also observed in rice [42].

Cytological studies have revealed that C49S and its derived lines C404S and C412S undergo normal meiosis [41,43]. YS-type, K-type, and P-type cytoplasmic male sterile wheat also exhibit normal meiosis, with the key abortive period occurring from the uninucleate to binucleate stage; abnormal tapetum degradation is the major cause of pollen abortion in these lines [44,45,46,47]. Thermo-sensitive genic male sterile wheat 4110S exhibits delayed programmed cell death (PCD) in the tapetum, leading to defective pollen exine formation and consequent male sterility [48]. In contrast, another thermo-sensitive genic male sterility wheat, Bainong (BNS), displays early degradation of the tapetum in sterile plants [49].

K64S, a key commercial sterile line used in southwestern China, is derived from C49S and exhibits consistent fertility performance in hybrid seed production. It has a 35-day sterile sowing window and a 24-day sterile period, nearly twice as long as that of C49S (unpublished data). However, its precise fertility alteration parameters and cytological mechanisms of pollen abortion remain uncharacterized. In this study, we investigated fertility alteration in K64S via controlled environment experiments to determine its sensitive developmental stages and critical temperature/photoperiod thresholds, complemented by histological analysis of anthers across developmental stages under fertile/sterile conditions, thereby understanding its fertility alteration characteristics and providing theoretical support for TPSGMS line breeding and hybrid seed production.

## 2. Results

### 2.1. The Fertility Alteration Sensitive Stage of K64S

The experiment was conducted at the Songming Experimental Station of YAAS. Meteorological data from the station showed that, during August–October 2022, the mean maximum temperatures were 26 °C (August), 25 °C (September), and 24 °C (October); the mean minimum temperatures were 17 °C, 18 °C, and 18 °C; and the mean temperatures were 21.5 °C, 21.5 °C, and 21 °C, respectively. Moreover, the average daylengths at the experimental site from August to October were 13.83 h, 13.62 h, and 13.50 h, respectively. These temperatures and daylengths were higher than the previous provisional critical thresholds (13.7 °C, 12.4 h) of fertility alteration identified through continual sowings from October to January under natural conditions [8]. This was further confirmed by the high pollen fertility rates of CK2 (K64S under natural conditions), which reached 95.61% and 96.04% (Table 1). Therefore, if low-temperature and short-daylength treatment were applied outside K64S’s fertility alteration-sensitive stage, the pollen fertility rate should remain close to that of CK2. Significant sterility would only occur if the low-temperature and short-daylength treatment coincided with the sensitive period.

K64S exposed to low-temperature and short-daylength treatment exhibited significant stage-specific variations in pollen fertility (Table 1). The S4 stage (pollen mother cell formation) demonstrated markedly lower pollen fertility rates (0.68% under 10 °C low temperature, 54.47% under 11 h short-day) compared to other developmental stages (≥93%). In contrast, Yangmai 33, an inbred cultivar, maintained high pollen fertility (>97%) across all treatments (Table 1), as did K64S under natural conditions (>95%). These results demonstrate that the fertility-sensitive window for both temperature and photoperiod in K64S spans from pollen mother cell formation through tetrad development. The 11 h short-day treatment induced only partial fertility reduction (54.47%), indicating a day-length of 11 h is too long to induce complete sterility under high-temperature conditions.

### 2.2. The Threshold of Fertility Alteration in K64S

Under constant daylength (14 h), the pollen fertility rate of K64S during the fertility alteration sensitive stage differed significantly across all temperature treatments and increased with rising treatment temperature (Table 2). At 12 °C, the pollen fertility rate was 0%, indicating complete sterility, which was the lowest among all temperature treatments. The pollen fertility rate was 4.51% at 14 °C, which did not differ significantly from that at 12 °C, also indicating high sterility. However, the pollen fertility rate was 12.45% at 14.5 °C, which was significantly higher than that at 12 °C and 14 °C, and exhibited partial fertility (pollen fertility rate > 10%). In contrast, the pollen fertility rates of Yangmai 33 were over 97% across all tested temperatures, with no significant differences. Therefore, the critical temperature for fertility alteration of K64S is 14–14.5 °C, with pollen sterility occurring below 14 °C and fertility recovery above 14.5 °C.

Under constant 21 °C, the pollen fertility rate of K64S during the fertility alteration sensitive stage differed significantly across photoperiod treatment and increased with longer daylength (Table 3). When K64S was exposed to 9 h daylength, it showed high sterility with a pollen fertility rate of 2.39%, the lowest among all treatments. Under a 9.5 h daylength, its pollen fertility rate was 16.51%, which was significantly higher than that under 9 h, indicating partial fertility. In contrast, the pollen fertility rate of Yangmai 33 was over 97% across all tested photoperiods, with no significant differences. Thus, the critical daylength for fertility alteration of K64S is 9–9.5 h, with pollen sterility occurring below 9 h and fertility recovery above 9.5 h.

In conclusion, the critical thresholds of fertility alteration in TPSGMS line K64S are 14–14.5 °C for temperature and 9–9.5 h for daylength.

### 2.3. Sterile K64S Demonstrated Normal Meiotic Progression

**Figure 1:**
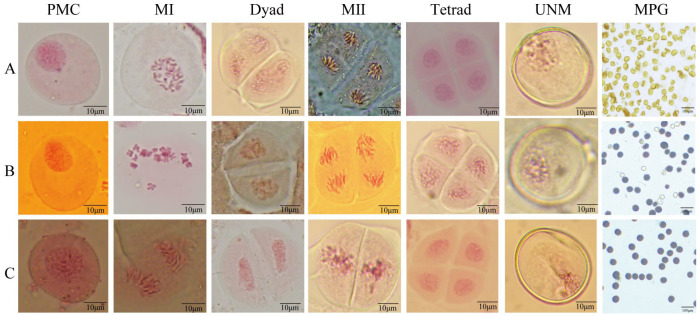
Cytological observation of meiosis in sterile and fertile K64S and Yangmai 33. (**A**): sterile K64S, (**B**): fertile K64S, (**C**): Yangmai 33. PMC: pollen mother cell, MI: metaphase I of meiosis, Dyad: dyad stage, MII: metaphase II of meiosis, Tetrad: tetrad stage, UNM: uninucleate microspore, MPG: Mature Pollen Grain stage (pollen stained by I_2_-KI). I_2_-KI typically stains normal fertile pollen dark brown or blue-black, whereas aborted pollen remains light-colored or unstained.

The meiotic stages in sterile K64S, fertile K64S, and Yangmai 33 are illustrated in Figure 1. The developmental progression from pollen mother cells to uninucleate microspores was consistent among fertile K64S, sterile K64S, and the inbred cultivar Yangmai 33, with no structural aberrations observed in sterile K64S plants. All three lines exhibited morphologically normal pollen mother cells with typical organelle distribution and nuclear organization at the pollen mother cell stage (Figure 1 PMC). Spindle fibers attached to chromosomal kinetochores and bivalent chromosomes aligned at the equatorial plates at metaphase I of meiosis (Figure 1 MI). Following meiosis I, chromosome decondensation, nucleolus reformation, nuclear envelope reestablishment, and cytokinesis produced two daughter cells at the dyad stage (Figure 1 Dyad). At metaphase II of meiosis, sister chromatids aligned at the equatorial plates with well-defined spindle apparatus in all genotypes (Figure 1 MII). All lines formed normal tetrads encapsulated within callose walls at the tetrad stage (Figure 1 Tetrad). No abnormalities were detected in microspore structure or nuclear positioning across genotypes at the uninucleate microspore stage (Figure 1 UNM).

Mature anthers (visually identified by yellow coloration) were assessed using I_2_-KI staining. Sterile K64S plants exhibited complete male sterility (0.00% pollen fertility) (Figure 1A (MPG)). In contrast, the pollen fertility rate of fertile K64S plants was 94.78 ± 1.03% (Figure 1B (MPG)), while Yangmai 33 had a pollen fertility of 96.42 ± 1.12% (Figure 1C (MPG)).

### 2.4. Pollen Abortion Occurs at the Late Uninucleate Microspore Stage

**Figure 2:**
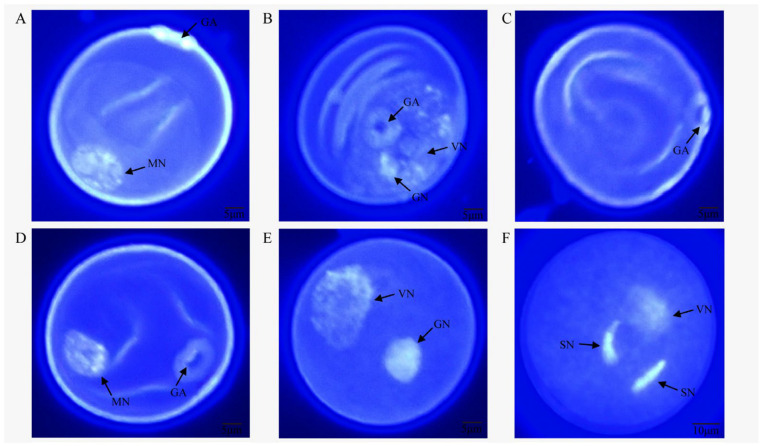
DAPI-based nuclear phenotyping of pollen developmental stages in sterile and fertile K64S. (**A**–**C**) indicate sterile K64S plants; (**D**–**F**) indicate fertile K64S plants; (**A**,**D**), uninucleate microspore, (**B**,**E**), binucleate microspore, (**C**,**F**), trinucleate microspore. GA: germinal aperture; MN: microspore nucleus; VN: vegetative nucleus; GN: generative nucleus; SN: sperm nuclei.

Results of DAPI staining of uninucleate, binucleate, and trinucleate microspores from sterile and fertile K64S plants are illustrated in Figure 2. Nuclear integrity remained unaffected during early microspore development, as both sterile and fertile K64S pollen contained distinct singular nuclei at the uninucleate microspore stage (Figure 2A,D). The critical fertility divergence occurred during the transition to binucleate microspores: aborted microspores contained a single undivided and diffuse nucleus (Figure 2B), contrasting with the dual-nuclear architecture in fertile pollen (Figure 2E). This indicates that microspore development terminates at the late uninucleate stage in sterile plants, leading to the formation of abnormal pollen grains. At the trinucleate microspore stage, sterile K64S pollen exhibited complete nuclear dissolution (Figure 2C), whereas fertile pollen displayed three distinct nuclei (two sperm nuclei and one vegetative nucleus) (Figure 2F). DAPI staining further confirmed that nuclear division and development are disrupted in sterile K64S pollen, directly resulting in male sterility.

### 2.5. Premature Tapetal Degradation Triggers Sporophytic Male Sterility in K64S

**Figure 3:**
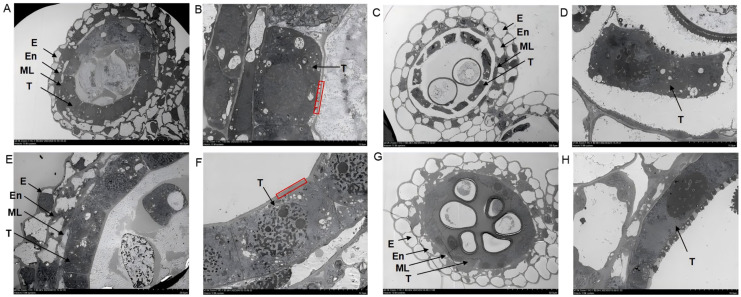
Observation of anthers by transmission electron microscopy in sterile and fertile K64S. (**A**,**B**), sterile K64S at pollen mother cell, (**C**,**D**), sterile K64S at uninucleate microspore; (**E**,**F**), fertile K64S at pollen mother cell, (**G**,**H**), fertile K64S at unicleate microspore. E: epidermis, En: endothecium, ML: middle layer, T: tapetum. The red boxes highlight the cytoplasmic-wall separation gaps.

Transmission electron microscopy (TEM) revealed that both sterile and fertile K64S plants exhibited a four-layered anther wall organization (epidermis, endothecium, middle layer, and tapetum) (Figure 3A,C,E,G). However, critical cytological differences were observed between sterile and fertile K64S. In the tapetum of sterile K64S, cytoplasmic condensation toward the locule occurred due to separation of the cytoplasm from the cell wall. No distinct nucleolar structures were observed, indicating nucleolar dissolution. Moreover, electron density in the nucleus decreased and the entire tapetal cell appeared darker (Figure 3B). These findings indicate that premature tapetal degeneration occurred as early as pollen mother cell stage in sterile K64S. By contrast, tapetal cells of fertile K64S contained distinct nucleoli within intact nuclei (Figure 3F). By the uninucleate microspore stage, the contour of the tapetal cells in sterile K64S had collapsed, with deeper staining and noticeable gaps between cells, indicating that tapetal cells had already reached the final stage of degeneration (Figure 3D). Meanwhile, fertile K64S tapetal cells exhibited chromatin margination (peripheral condensation), nucleolar disappearance and nuclear membrane invagination at the uninucleate microspore stage, indicating that the tapetal cells just entered the early stage of degeneration (Figure 3H). Premature initiation of tapetal degeneration in sterile K64S during microsporogenesis leads to subsequent energy and nutrient deprivation, representing the primary cause of pollen abortion.

## 3. Discussion

In environment-sensitive genic male sterility-based two-line hybrid rice and wheat, the fertility alteration sensitive stage and fertility alteration thresholds are key fertility parameters for assessing a sterile line’s application potential [50]. These parameters can be identified both in natural and controlled conditions. However, under natural conditions, it is difficult to decouple the effects of light and temperature on the fertility of sterile lines. Moreover, temperature and light conditions vary greatly among regions, years, and even within a single day, which brings challenges for identifying and in-depth studying the fertility nature and genetic basis of environment-sensitive genic male sterility; therefore, research under artificially controlled conditions is essential, which also enables provision of a general guide to how to use the sterile line [51]. This study shows that the fertility alteration thresholds of K64S are 14–14.5 °C for temperature and 9–9.5 h for daylength during the fertility alteration sensitive period from pollen mother cell formation to tetrad; thus, K64S is 4–4.5 °C higher than its progenitor line C49S in temperature threshold, 1.5–2 h shorter in daylength threshold [52]. Considering the daylength with light intensity ≥ 10,000 lx in Yunnan plateau is shorter than eight hours during the whole wheat growing season from November to next April, K64S, with higher temperature threshold, will have wider adaptability in Yunnan Province compared with C49S (Table S1).

Although the critical values of K64S fertility alteration obtained in this study are specific to the controlled constant conditions, these values specify the boundary conditions of temperature and daylength for the use of K64S to produce high-purity hybrid seeds under fluctuating natural conditions. Based on Yunnan’s spring climate profile (Table S1) and the critical values of K64S, we can propose following strategies for hybrid seed production or sterile line multiplication: (1) when daily mean temperature during the sensitive stage ≤ 14.5 °C, K64S maintains high sterility (>95%) regardless of daylength, it is safe for hybrid seed production; (2) when temperature ≥ 18 °C (multiplication conditions), daylength has a major effect on fertility: ≥11 h gives full fertility (<5% sterility) and ≤9 h gives partial sterility (ca. 40% fertility); (3) for temperatures between 14.5 °C and 18 °C, reliable predictions are not available, indicating both hybrid seed production and sterile line multiplication should be avoided. Thus, temperature is the dominant factor for hybrid seed production, while daylength matters mainly under high-temperature for sterile line multiplication. In addition, when K64S is used for hybrid seed production in a given region, identification of its fertility parameters under local natural conditions is necessary by sowing date experiment, as exemplified by our previous report [39]. Future factorial experiments are needed for a quantitative model.

To minimize asynchrony, we used uniform plants, morphological indicators, and focused on the two middle spikelets of the main stem. Asynchrony is inherent, but our staging was based on the four basal florets of these two spikelets—the earliest in the spike. When these reached S4, other florets were slightly less advanced (still between S3 and S4), as supported by >93% fertility in earlier stages. Thus, the sensitive stage identified (S4) precisely defines the developmental window of peak sensitivity. Importantly, both staging and fertility assessment were performed on the same two middle spikelets, so the reported peak window accurately reflects the most synchronous part of the spike, and the slight delay elsewhere does not affect the threshold. We acknowledge that using the entire spike would give a broader apparent window, but we deliberately restricted assessment to the two middle spikelets to obtain a precise stage-specific reference. In contrast, in the field of hybrid seed production, the field fertility sensitive period of K64S will be far wider than the result reported here because the sterile line is a population—including all tillers of a plant and all spikes in a field. The first floret of the earliest spike may enter the sensitive stage 1–2 weeks earlier than the last floret of the latest tiller. In this situation, the fertility sensitive period together with the fertility alteration thresholds identified in controlled conditions, will be used as a guide to screening suitable location and optimum sowing dates, to couple the field fertility sensitive period with local low-temperature months, consequently guaranteeing the production of high-purity hybrid seeds [39].

The duration of the fertility alteration sensitive stage in most widely used TPSGMS lines is longer than that of K64S. For example, the sensitive stage of C49S and K78S extends from booting to heading stage [39], while that of BS20 [53] and BS210 [54] extends from anther septum formation to uninucleate microspore formation stage, and that of BNS extends from floret primordium to pistil and stamen primordium [55]. Moreover, these environment-sensitive genic male sterile lines have different fertility alteration thresholds, which facilitates the application of two-line hybrid wheat across different ecological zones in China.

The tapetum, as the innermost layer of the anther wall, is in direct contact with developing gametophytes. It provides enzymes for the release of microspores from tetrads and nutrients for pollen development during transition from microspores to mature pollen grains [56,57]. Production of functional pollen grains depends critically on the timely initiation and progression of tapetal degeneration. In this study, we found that tapetal degeneration in sterile K64S initiates at the pollen mother cell stage, one or two stages earlier than in fertile K64S. This pattern in K64S contrasts with that of its progenitor C49S, which exhibits delayed tapetal degradation [58]. It appears that premature tapetal degeneration disrupts nutrition and energy supply from the early stage of microspore development, consequently resulting in complete pollen abortion in K64S (Figure 1A). This temporal shift in tapetal degeneration implies distinct molecular triggers for sterility despite the shared genetic ancestry. Similarly, premature tapetal PCD as a main cause of microspore abortion has also been reported in Bainong thermo-sensitive male sterile (BNS) wheat, photoperiod-sensitive male sterile rice Nongken 58S [49,59,60], and sterile wheat induced by chemical hybridization agent SQ-1 [61], suggesting that premature tapetal PCD is a common cause of pollen abortion across different sterility systems.

In summary, pollen abortion in K64S occurs earlier and more completely than in its progenitor C49S, resulting in different hybrid seed production strategies. This indicates that different male sterile wheat lines can have distinct cytological mechanisms of pollen sterility.

Quantitative TEM morphometric analysis (e.g., measuring cytoplasmic condensation or organelle integrity) was not performed due to the limited number of anthers suitable for sectioning at each stage. It will be addressed in future studies with larger samples.

The present study focuses on cytological mechanism, and the molecular regulatory network (e.g., candidate genes controlling tapetal PCD timing) remains unknown. Comparative transcriptomics between K64S and its progenitor C49S is underway as a logical next step. In addition, future factorial experiments with combined temperature and photoperiod treatments will be conducted to obtain more complete interaction data, enhancing theoretical support for hybrid wheat breeding and production.

## 4. Materials and Methods

### 4.1. Plant Materials

The thermo-photo-sensitive genic male sterile (TPSGMS) wheat line K64S was provided by the Food Crops Research Institute, Yunnan Academy of Agricultural Sciences (YAAS), Kunming, China. The inbred cultivar Yangmai 33 was obtained from the Jiangsu Lixiahe Regional Institute of Agricultural Sciences, Yangzhou, China.

### 4.2. Fertility Alteration Analysis of K64S

#### 4.2.1. Plant Cultivation

K64S and Yangmai 33 were sown in 26 cm × 23 cm pots with 10 seeds per pot. When plants reached the jointing stage, five uniformly developed seedlings were retained per pot, and plants were grown at the Songming Experimental Station (25°35′ N, 103°38′ E; altitude 1997 m) of YAAS.

#### 4.2.2. Identification of Sensitive Stages of Fertility Alteration

The experiment was conducted from 1 August to 31 October 2022. When plants reached specific developmental stages (S1, floret primordium differentiation; S2, stamen and pistil primordium differentiation; S3, anther septum formation; S4, pollen mother cell formation; S5, uninucleate microspore formation; S6, binucleate microspore formation; and S7, trinucleate microspore formation), K64S and Yangmai 33 (control 1, CK1) at identical developmental stages were transferred to growth chambers (RXZ-1000B, Nibo Jiangnan Instrument Factory, Ningbo, China) and maintained under treatment until entering the next developmental stage (with the exception of trinucleate microspore formation). For each treatment, 25 plants (in five pots) were treated. The main-stem spike of each plant was used for developmental stage identification, and its two middle spikelets were used for fertility assessment. K64S grown under natural conditions served as an additional control (CK2). After chamber treatment, plants were returned to natural conditions and grown until flowering for pollen fertility assessment.

Based on our preparatory experiment conducted during the period from August to October 2021 with K64S, the two chamber treatment conditions were set as 10 °C (low temperature) with a 14 h light/10 h dark and 21 °C (high temperature) with an 11 h light/13 h dark, respectively, maintaining constant light intensity at 10,000 lx and relative humidity at 75 ± 5% across all treatments.

In the same preparatory experiment, we examined >100 main-stem spikes of K64S to calibrate the relationship between morphologic indicators and interior spike differentiation (S1–S3) or microsporogenesis stages (S4–S7), by recording leaf development progress and observing the two middle spikelets of a spike with a stereo-microscope (S1–S3), or examining four anthers—one from each of four basal florets in the two middle spikelets with a microscope (S4–S7). The relationship was established as follows: S1 ≈ 5 leaves + 3/5 emerged flag leaf; S2 ≈ 6 leaves + 1/2 flag leaf; S3 ≈ 7 leaves + 1/2 flag leaf; S4 ≈ pulvinus distance at 2–8 cm; S5 ≈ pulvinus distance at 8.5–9.5 cm; S6 ≈ awn emerging; S7 ≈ fully emerged spike (unpublished data). In this study, when morphological indicators of the main-stem spikes suggested the plants were approaching the target stage before starting or terminating chamber treatments, five plants were randomly sampled from five pots to examine their interior developmental stages using methods above. During treatment, morphological indicators were monitored daily. When treated plant grew to the early flowering stage, pollen fertility was assessed using the two middle spikelets of main-stem spike of each plant. For each treatment, the pollen fertile rate was calculated as the mean of all plants.

#### 4.2.3. Experimental Design for Threshold Determination

Temperature and daylength thresholds for fertility alteration were determined via controlled environment experiments conducted from 1 November 2022 to 30 January 2023. K64S plants at the fertility-sensitive stage were subjected to two sets of treatments: (1) four temperature regimes: 12 °C, 14 °C, 16 °C, and 18 °C with a constant 14 h light/10 h dark photoperiod, and (2) four photoperiod conditions: 9 h, 10 h, 11 h, and 12 h light with a constant temperature of 21 °C. Both treatments sets maintained a constant light intensity of 10,000 lx (LED) and 75% relative humidity. After identifying the approximate primary threshold, finer 0.5 °C thermal gradients and 0.5 h photoperiod gradients were implemented within the critical range to refine the threshold values.

Due to the constraints of growth chamber capacity, this study employed single-factor designs (varying temperatures under constant long-day; varying photoperiods under constant high-temperature). Full factorial combinations of temperature and photoperiod were not tested.

#### 4.2.4. Pollen Fertility Assessment

When the main-stem spike of treated plant begins to flower, anthers from two middle spikelets were collected for pollen staining with 1% I2-KI solution. Pollen grains were classified as normal (fully stained and spherical) or aborted. Aborted pollen was further categorized to typical abortion (unstained and irregular shape), circular abortion (unstained and spherical shape), and stained abortion (partially stained).

Pollen fertility rate (%) = (Number of normal pollen/Total number of observed pollen) × 100

The pollen fertility rate < 10% was classified as sterile [62].

#### 4.2.5. Statistical Analysis

All data were analyzed using DPS V18.10 software.

### 4.3. Cytological Analysis of K64S Sterility

#### 4.3.1. Fertile/Sterile Plant Induction

K64S plants at the fertility alteration sensitive developmental stage were exposed to contrasting environmental regimes in controlled growth chambers to induce fertility under high-temperature and long-day conditions (21 °C, 14 h light/10 h dark) and sterility under low-temperature and short-day condition (12 °C, 10 h light/14 h dark). Upon reaching the uninucleate microspore stage, all plants were maintained under uniform conditions (20 °C, 14 h light/10 h dark, 75% relative humidity) until trinucleate microspore formation.

#### 4.3.2. Observation of Meiotic Stages

Anthers from fertile/sterile K64S and Yangmai 33 plants were collected during the developmental stages from pollen mother cell to uninucleate microspore. Samples were fixed in Carnoy’s solution (3:1 *v*/*v* 95% ethanol: glacial acetic acid) for 3 h at 4 °C and then stored in 95% ethanol at 4 °C. Anthers were stained with acetocarmine; microspores were gently tapped onto a glass slide, and observed under a microscope (Olympus CX43, Evident, Guangzhou, China).

#### 4.3.3. Cytological Stage of Abortion

Anthers at uninucleate, binucleate, and trinucleate developmental stages were fixed as described above. Microspores were gently released, stained with DAPI (4′,6-diamidino-2-phenylindole) (10 μg/mL) for 10 min and observed using a confocal microscope (Olympus BX53, Evident, Guangzhou, China).

#### 4.3.4. Transmission Electron Microscopy Analysis

Anthers at four developmental stages (pollen mother cell, dyad, tetrad, and uninucleate microspore) were fixed in FAA (50% ethanol:formalin:acetic acid = 18:1:1) and processed for transmission electron microscopy at the Biotechnology and Genetic Germplasm Resource Research Institute, YAAS.

## Figures and Tables

**Table 1:** Pollen fertility rate of K64S at different developmental stages under low temperature and short photoperiod.

Treatment Stages	Treated K64S %		CK1 %		CK2 %	
	10 °C/14 h	21 °C/11 h	10 °C/14 h	21 °C/11 h		
S1	94.15 ± 1.02 a	94.60 ± 1.08 a	97.87 ± 1.62 a	98.2 ± 1.08 a	95.61 ± 1.34	96.04 ± 1.20
S2	94.48 ± 0.98 a	93.78 ± 1.13 a	97.56 ± 1.23 a	98.62 ± 1.22 a		
S3	93.37 ± 1.11 a	93.84 ± 0.97 a	98.32 ± 0.97 a	98.13 ± 0.98 a		
S4	0.68 ± 0.12 b	54.47 ± 1.04 b	97.26 ± 1.12 a	97.44 ± 1.04 a		
S5	93.81 ± 0.87 a	94.10 ± 1.22 a	98.11 ± 1.23 a	98.43 ± 1.06 a		
S6	94.42 ± 1.22 a	94.87 ± 1.32 a	97.66 ± 0.99 a	98.47 ± 1.14 a		
S7	94.12 ± 0.89 a	94.14 ± 1.26 a	98.42 ± 0.98 a	97.14 ± 1.13 a		

S1, floret primordium differentiation stage; S2, stamen and pistil primordium differentiation stage; S3, anther septum formation stage; S4, pollen mother cell formation stage; S5, uninucleate microspore formation stage; S6, binucleate microspore formation stage; S7, trinucleate microspore formation stage. The same letter is not significantly different based on the least significant difference (LSD) at *p* ≤ 0.05; different letters indicate significant differences at *p* ≤ 0.05.

**Table 2:** Pollen fertility rate of K64S under a 14 h photoperiod and different temperatures.

Temperature	K64S	Yangmai 33
12 °C	0.00 ± 0.01 e	97.02 ± 1.78 a
14 °C	4.51 ± 0.46 e	98.55 ± 0.96 a
14.5 °C	12.45 ± 1.22 d	98.12 ± 0.94 a
15 °C	21.86 ± 2.07 c	98.31 ± 1.05 a
16 °C	36.03 ± 1.82 b	98.46 ± 1.12 a
18 °C	94.12 ± 1.56 a	99.03 ± 0.62 a

The same letter is not significantly different based on the least significant difference (LSD) at *p* ≤ 0.05; different letters indicate significant differences at *p* ≤ 0.05.

**Table 3:** Pollen fertility rate of K64S under constant 21 °C and different daylengths.

Photoperiod	K64S	Yangmai 33
9 h	2.39 ± 0.12 d	97.49 ± 1.21 a
9.5 h	16.51 ± 1.84 c	98.38 ± 1.06 a
10 h	21.49 ± 1.43 c	97.95 ± 2.18 a
11 h	54.47 ± 2.01 b	98.13 ± 1.76 a
12 h	94.10 ± 2.22 a	97.64 ± 1.47 a

The same letter is not significantly different based on the least significant difference (LSD) at *p* ≤ 0.05; different letters indicate significant differences at *p* ≤ 0.05.

## Data Availability

The original contributions presented in this study are included in the article/Supplementary Material. Further inquiries can be directed to the corresponding authors.

## Supplementary Materials

The following supporting information can be downloaded at: https://www.mdpi.com/article/10.3390/plants15121774/s1, Table S1: Monthly mean temperatures during early spring at multiple sites across Yunnan Province, southwest China (2016–2025).

## Abbreviations

The following abbreviations are used in this manuscript:

TPSGMS	thermo-photo-sensitive genic male sterile
DAPI	4′,6-diamidino-2-phenylindole
PCD	programmed cell death
