# Supplementary material for: Fertility Alteration Characteristics and Cytological Mechanisms of Pollen Abortion in Thermo-Photo-Sensitive Genic Male Sterile Wheat K64S
Source: Plants (Basel). 2026 Jun 9;15(12):1774. doi: 10.3390/plants15121774 (PMC13306847; doi:10.3390/plants15121774)
Supplement: Supplementary file 1 [file plants-15-01774-s001.zip › plants-4343537-supplementary.pdf]

Supplementary Table S1 Monthly mean temperatures (°C) during early spring at multiple sites across Yunnan Province, southwest China (2016–2025).

Location	Altitude (m)	Critical Month	Temp. Range (°C)	2016	2017	2018	2019	2020	2021	2022	2023	2024	2025
**Wenshan**	1500	February	9.5-14.0	11.5	13.5	11.5	13.0	14.0	9.5	12.0	10.0	12.5	9.5
		March	10.0-17.5	10.0	13.0	12.5	17.5	14.5	14.5	11.0	15.0	15.5	12.5
**Baoshan**	1700	February	9.0-13.0	12.0	13.0	12.5	12.5	12.5	12.5	9.0	12.5	11.0	11.0
		March	14.0-16.0	14.5	14.0	15.0	15.0	16.5	16.0	16.0	14.5	14.0	16.0
**Chuxiong**	1800	February	10.0-13.5	11.0	12.5	12.0	13.5	12.0	12.0	10.0	13.5	13.0	11.0
		March	14.0-18.0	15.5	14.0	15.0	16.0	17.0	16.5	18.0	16.5	17.0	15.5
**Qujing**	1850	February	6.0-14.0	6.0	10.5	9.5	14.0	11.5	10.5	8.0	12.0	13.0	10.0
		March	12.5-15.5	13.5	12.5	14.5	14.5	15.5	15.5	15.5	15.0	15.5	13.0
**Kunming**	1950	February	9.0-14.0	9.0	11.0	9.5	12.5	11.0	12.0	9.5	12.5	14.0	11.0
		March	13.0-16.5	14.5	13.0	13.5	14.0	16.0	16.0	16.5	15.5	16.5	15.0
**Dali**	2000	February	7.5-11.5	10.0	11.5	11.0	11.5	10.5	11.0	8.0	11.5	10.0	7.5
		March	12.5-15.0	13.0	12.5	14.0	13.5	14.5	14.5	15.0	14.0	14.5	13.0
**Lijiang**	2400	February	5.0-9.5	9.0	9.5	9.0	8.5	8.0	8.5	6.0	9.5	8.0	5.0
		March	10.0-15.0	12.0	11.0	11.5	11.0	12.5	12.5	15.0	12.5	13.0	10.0
